# Establishment of Dissolution Test Method for Multi-Components in Traditional Chinese Medicine Preparations Based on In Vitro–In Vivo Correlation

**DOI:** 10.3390/ph17081065

**Published:** 2024-08-14

**Authors:** Pengcheng Guo, Qizheng Wang, Xiaoqiang Xiang, Yufeng Zhang, Yue Pan, Zhong Zuo, Jianxin Wang

**Affiliations:** 1 Department of Pharmaceutics, School of Pharmacy, Fudan University & Key Laboratory of Smart Drug Delivery, Ministry of Education, Shanghai 201203, China; 20111030046@fudan.edu.cn (P.G.); pharma_wang@126.com (Q.W.); panyue1107@126.com (Y.P.); 2 Department of Clinical Pharmacy and Pharmacy Administration, School of Pharmacy, Fudan University, Shanghai 201203, China; xiangxq@fudan.edu.cn; 3 School of Pharmacy, Faculty of Medicine, The Chinese University of Hong Kong, Shatin, New Territories, Hong Kong SAR, China; zhangyf@cuhk.edu.hk; 4 Institute of Integrated Chinese and Western Medicine, Fudan University, Shanghai 200040, China

**Keywords:** quality control, dissolution condition, multi-component integration, Yuanhu Zhitong tablet, biopharmaceutic classification system

## Abstract

In this study, a multi-component integrated dissolution evaluation system of Yuanhu Zhitong tablets (YZTs) was established based on in vitro and in vivo correlation (IVIVC). The dissolution tests of five quality markers (Q-markers), including tetrahydropalmatine, α-allocryptopine, protopine, corydaline, and byakangelicin, in YZTs were conducted under different dissolution conditions, and pharmacokinetic studies were performed in beagle dogs to construct a correlation model using numerical deconvolution. The data of the five ingredients were integrated in vitro and in vivo according to the biopharmaceutical classification system (BCS) to establish an IVIVC integrating multiple Q-markers. The dissolution media with the best correlation of components were obtained and validated. The results showed that all five components were classified as BCS I compounds, and α-allocryptopine, byakangelicin, tetrahydropalmatine, and corydaline showed good correlation in the paddle method, 75 rpm, with dissolution media of artificial gastric fluid, acetate buffer, acetate buffer and 0.1 M HCl, respectively. Protopine showed good correlation in the paddle method, 100 rpm, with dissolution media of 0.1 M HCl. The integrated BCS I Q-markers showed the best correlation in the medium of acetate buffer. The multi-component integrated dissolution evaluation system established in this experiment accurately predicted the pharmacokinetic data of YZTs by verifying the media, which can be used for the quality control of YZTs. The present study provides an effective and promising strategy for the dissolution evaluation for traditional Chinese medicine preparations.

## 1. Introduction

Traditional Chinese medicine preparations (TCMPs) have been used for thousands of years and have a huge market in China. Most of them are oral solid preparations, including tablets, capsules, granules, etc. It is well known that the efficacy and safety of oral administration drugs depend on their disintegration, dissolution, and absorption processes in the body. For oral solid dosage forms, it is necessary to measure the dissolution in simulated gastrointestinal fluids to evaluate the degree and speed of drug release from the formulation in gastrointestinal tract, and to evaluate the consistency of its in vivo fate and efficacy by detecting the similarity of in vitro dissolution of different sources or batches of preparations [1,2]. However, at present, the quality control of TCMP is mainly to determine the content of one or several index components, but the in vitro dissolution and in vivo absorption processes of these components are hardly studied and controlled [3]. Since the preparation is the final form of clinical medicine, if the active ingredients of traditional Chinese medicine cannot be dissolved and absorbed from the preparation, it will not be able to exert the intended therapeutic effect. Therefore, the dissolution of TCMP is an important indicator related to its quality stability, safety, and effectiveness. Therefore, it is necessary and urgent to study and establish a scientific and feasible evaluation system for the dissolution of TCMP solid preparations according to their specific characteristics, so as to improve the internal quality of TCMPs and ensure the consistency of the quality and efficacy of them [4,5].

There are many reports on the dissolution of TCMP, but most of them just take a single-index component to evaluate the dissolution. For example, the European Union stipulates that for immediate-release herbal medicinal products containing standardized extracts, the dissolution of analytical markers should be studied [6]. However, the vast majority of TCMPs contain multiple medicinal materials, and their clinical efficacy de-pends on the comprehensive effect of multiple components in the body. In an effort to explore dissolution evaluation methods tailored to active components of TCMPs, re-searchers have engaged in extensive work and proposed various research approaches, including simultaneous determination of multiple active ingredients [7], substance group determination methods [8,9], absorbance area methods [10,11], fingerprinting [12], and bioequivalence methods [13].

Although these methods reflect the dissolution characteristics of multiple components of TCMP to a certain extent, two key issues are usually ignored. Firstly, the rationality of the in vitro dissolution method is based on the high correlation with the in vivo absorption process, that is, it must have in vitro–in vivo correlation (IVIVC), which is a prerequisite for the use of in vitro dissolution to reflect the absorption and even efficacy of drugs in vivo. However, IVIVC was rarely studied to evaluate the significance of dissolution of TCMP. Secondly, the selected compounds for dissolution test usually were not active components, only because they are easy to detect. Thus, a more scientific and reasonable system should be developed for the dissolution test of multiple components in TCMP.

The Yuanhu Zhitong tablet (YZT) is a TCMP prepared from the extract and powder of *Corydalis rhizoma* and *Angelica dahurica* [14], which are often added with some pharmaceutical adjuvants such as magnesium stearate, talc powder, and sodium carboxymethyl starch as dissolving agents or lubricant in pharmaceutical factories. YZTs have been widely used to treat stomach pain, hypochondriac, neuropathic headache, traumatic headache, and dysmenorrhea in China [15]. Since its clinically validated effectiveness and safety, it has been officially included in the Pharmacopoeia of the People’s Republic of China (ChP) since 1985 [16,17]. The main active components of YZTs are alkaloids and coumarins [18,19], and 65 alkaloids and 97 coumarins were identified in YZTs [20]. The former, including dcorydoline, berberine, and tetrahydropalmatine, exhibits diverse functions such as analgesia, improvement of microcirculation, dilation of coronary blood flow etc. [21,22], and the latter, including imperatorin and isoimperatorin, can enhance blood circulation by reducing malondialdehyde and nitric oxide levels, thus alleviating pain. Coumarins also contribute to increased alkaloid solubility and bioavailability [23,24]. Studies revealed that 21 of the compounds can be absorbed into the bloodstream, and 17 can permeate the blood–brain barrier [25,26]. Although YZTs have been applied in clinic for many years and manufactured by many companies, there is no dissolution test in their quality standard in ChP. Previous studies have explored the dissolution condition of YZTs. For example, Zhu et al. measured the dissolution of tetrahydropalmatine from YZTs from various manufacturers in different media to assess drug quality [27]. Nevertheless, one compound cannot reflect the dissolution of active components from the tablet, and the dissolution conditions established may not correlated with in vivo drug release and absorption.

In 2016, Liu introduced the concept of quality markers (Q-markers) to evaluate and standardize and the quality of TCMP [28]. Through a comprehensive analysis that encompassed fingerprinting [23,29], pharmacokinetics [30], drug metabolism [31], network pharmacology [26], systematic biology, pharmacodynamics, and other methods, a limited set of active ingredients was identified to represent the overall properties and efficacy of TCMP. Q-markers have garnered widespread research attention and recognition for the characterization of Chinese herbs and TCMP, and applied in many drugs [32,33]. Q-markers have been supposed to be a revolutionary innovation for the quality control of TCMP. Liu and his colleagues have successfully identified the Q-markers for YZTs, which are tetrahydropalmatine, α-allocryptopine, protopine, corydaline, and byakangelicin [34]. The results provide a solid foundation for dissolution study by taking Q-markers as the indexes, which can reflect the effect of the original preparation.

In vitro–in vivo correlation (IVIVC) serves as a critical quality control tool [35]. Dissolution methods established based on the IVIVC model can accurately predict in vivo drug bioavailability and serve as a surrogate for bioequivalence. The establishment of IVIVC models typically involves two steps. Firstly, it entails obtaining the in vivo absorption curve through the deconvolution of pharmacokinetic curves. Then, it establishes a correlation between the in vitro dissolution curve and the in vivo absorption curve using an appropriate equation. Commonly used deconvolution algorithms include Wagner–Nelson, Loo–Riegelman, and numerical integration methods, among others. This method has been applied to various formulations [36,37,38] such as mesalazine enteric-coated tablets [39], immediate-release ibuprofen tablets [40], long-acting release formulations of progesterone [41], telmisartan [42], and pitavastatin calcium [43]. It is gradually being adopted for in vivo use in TCMP compound formulations. For instance, Wang [44] and Li [45] established IVIVC models for intragastric floating iritin sustained-release patches and andrographolic tablets to evaluate the quality of the preparations. However, most studies have only considered a few key index ingredients, and this approach cannot comprehensively represent the overall properties of multi-component TCMP compound formulations.

Unlike single-compound chemical drugs, interactions among various quality marker components may exist during the absorption process, potentially affecting their in vivo pharmacokinetics. Prior to establishing IVIVC models, it is essential to rationally integrate in vitro dissolution data and in vivo pharmacokinetic data based on the biopharmaceutical classification (BCS) of each active ingredient. Since the compounds in the same BCS class have similar dissolution and absorption characteristics, in order to simplify the IVIVC calculation and dissolution evaluation, the individual dissolution profiles of the Q-markers within a class are integrated into a single dissolution profile based on their content in the tablet, and the plasma concentrations were summed up and integrated into a multi-component concentration–time curve. However, it is important to note that BCS Class I and III components are highly soluble, and their dissolution is not affected by the dissolution medium, allowing for direct integration. In contrast, the pKa physical properties of BCS Class II and IV components significantly impact their dissolution in different media [46]. This discrepancy can result in differences between integrated in vivo and in vitro data and the true value. Tsume et al. [47] proposed an extension of the BCS classification which includes subclassifications or subfamilies within Classes II and IV: Weak acid (a), weak base (b), and neutral (c). This classification is based on the physical properties of each drug; components within the same subclassification exhibit similar dissolution properties. Therefore, it is necessary to study the subclassifications of BCS Classes II and IV before integrating in vivo and in vitro data, with components from the same subclassification being integrated for both types of data.

In order to explore the scientific in vitro dissolution method of TCMPs containing a variety of active ingredients, in the present study, YZT was chosen as a model drug, and Q-markers were used as the active ingredients, to establish an in vitro dissolution method based on in vivo absorption kinetics. This approach aims to construct multi-component integrated IVIVC models, thereby creating an external dissolution evaluation method that accurately reflects the in vivo behavior of the TCMP formulation.

## 2. Results and Discussion

### 2.1. Solubility of Q-Markers in YZTs

By measuring the concentration of each ingredient in different media, the solubility results are shown in Table 1. The maximum dose (M_0_) was determined by measuring the content of each component in a single dose. The D_0_ calculated from the solubility results are shown in Table 1. Although the solubility values of tetrahydropalmatine, corydaline, protopin, and byakangelicin are poor, the content of each component in the tablet is relatively low; therefore, the five components meet the requirement of D_0_ less than 1, indicating that they were all highly soluble drugs in YZTs.

### 2.2. Permeability of Q-Markers in YZTs

The P_eff_ of five components in the transport experiments results were greater than 1 × 10^−6^ cm/s, which means that all of them have good permeability and belong to high-permeability compounds. The permeability results were consistent with those obtained by Chen [33] using the everted intestinal sac model. 

Based on the permeability and solubility results, the BCS of all the five components was determined (Table 2) to be Class I. which simplifies the difficulty of data integration and improves the likelihood of establishing multi-component IVIVC.

### 2.3. Dissolution of Q-Markers from YZTs

In the dissolution test, the volume of the dissolved medium should be sufficient to ensure a reasonable leakage condition. Generally, it is assumed that the test volume is 3 times the saturated volume. According to the measured solubility results, the solubility of tetrahydropalmatine, corydaline, protopin, α-allocryptopine, and byakangelicin in water were 18.87 ± 1.57 μg/mL, 5.03 ± 0.85 μg/mL, 72.12 ± 9.54 μg/mL, 2376.09 ± 521.11 μg/mL, and 35.90 ± 5.85 μg/mL, respectively. Based on the above results, the maximum daily dose of dissolution (3749.66 μg, 144.75 μg, 2843.44 μg, 1375.21 μg, 4174.73 μg) requires much less than 900 mL of media. In our present study, 900 mL dissolution medium could meet the requirement of leakage conditions.

**Figure 1:**
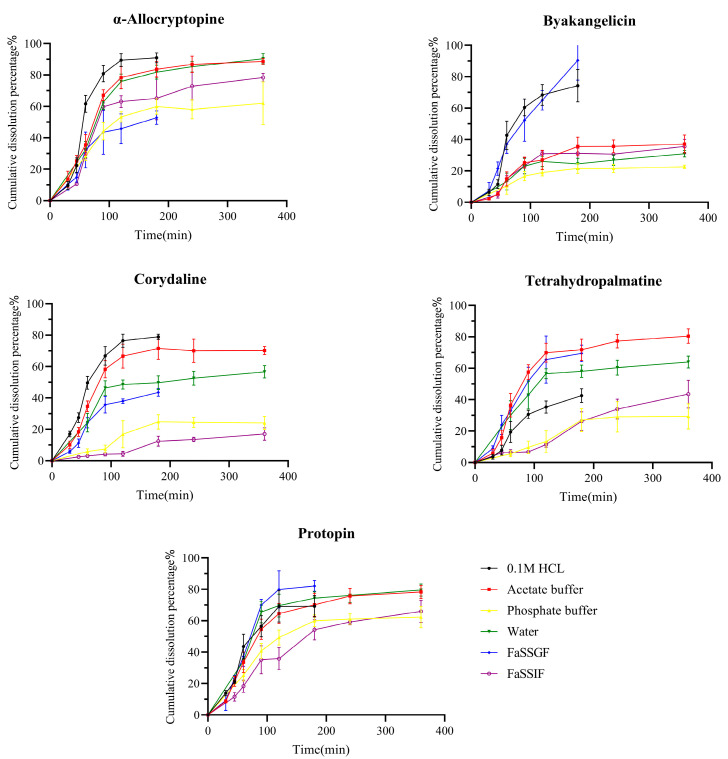
Dissolution profiles of protopin, α-allocryptopine, byakangelicin, tetrahydropalmatine, corydaline in YZTs in different media (0.1 M of HCl, acetate buffer, phosphate buffer, FaSSIF-V2, and FaSSGF *n* = 6).

The dissolution study results of the tetrahydropalmatine, corydaline, protopin, α-allocryptopine, and byakangelicicn are shown in Figure 1. Protopine exhibited the highest dissolution in FaSSGF, with relatively poor dissolution extent in phosphate buffer and simulated intestinal fluid. α-allocryptopine rapidly dissolved in 0.1 M of HCl, acetate buffer, and water, but released less than 60% within 6 h in phosphate buffer and artificial intestinal fluid. Tetrahydropalmatine showed good dissolution efficiency in pH 4.5 acetate buffer and FaSSIF, while corydaline performed better at lower pH. Considering these results, protopine, corydaline, tetrahydropalmatine, and α-allocryptopine showed an increasing trend in dissolution efficiency as the pH of the dissolution medium decreases. The solubility results of these components suggested lower solubility in pH 6.8 phosphate buffer, thus the dissolution trend aligns with the solubility results of each component. Byakangelicin dissolved well in simulated gastric fluid and 0.1 M of HCl, with less than 40% released in all other dissolution media. Although the five Q-markers fall within BCS I with high solubility and high permeability, the dissolution results of the five Q-markers do not reach 100%. The reason is that the complex raw materials of TCMPs, such as medicinal powder and extract, delay the disintegration time of the preparation and reduce the dissolution rate of the active ingredient, resulting in the reduction in the dissolution of components.

### 2.4. UPLC-MS/MS Method Validation for Blood Concentration Determination of Q-Markers

**Figure 2:**
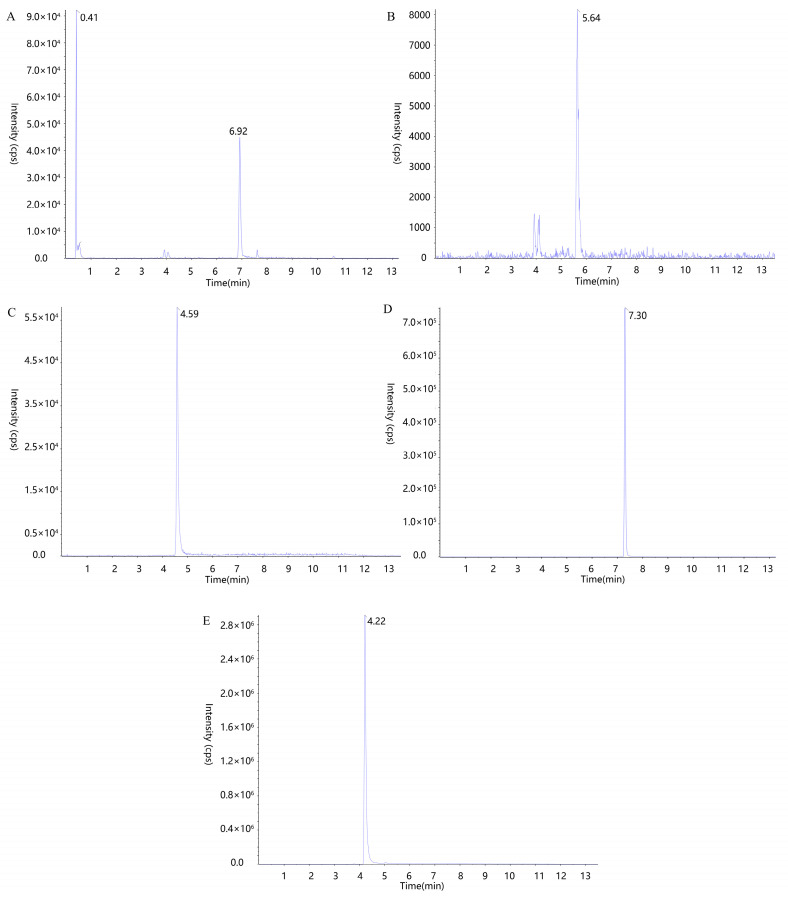
UPLC-MS/MS chromatograms of tetrahydropalmatine (**A**) 6.92 min and corydaline (**A**) 0.41 min, byakangelicin (**B**) 5.64 min, α-allocryptopine (**C**) 4.59 min, protopin (**E**) 4.22 min, and IS (**D**) 7.30 min of YZTs in plasma.

The UPLC-MS/MS chromatograms of five Q-markers, i.e., tetrahydropalmatine, corydaline, protopin, α-allocryptopine, and byakangelicin, in the plasma are shown in Figure 2. The standard curves of each component, obtained by measuring the peak areas of the standard samples at different concentrations (Table S1), indicate good correlations in the range and high sensitivity of the analytical method. The recoveries of the five components ranged from 85.39% to 110.92%, and the precision ranged from 1.27% to 11.44%, with detailed results in Tables S2 and S3. The stability results in Table S4 show that the components could keep stable for 24 h at 20 °C, 6 h in autosampler for liquid chromatography (6 °C) and freeze–thaw thrice.

### 2.5. Pharmacokinetics of Q-Markers in YZTs

**Figure 3:**
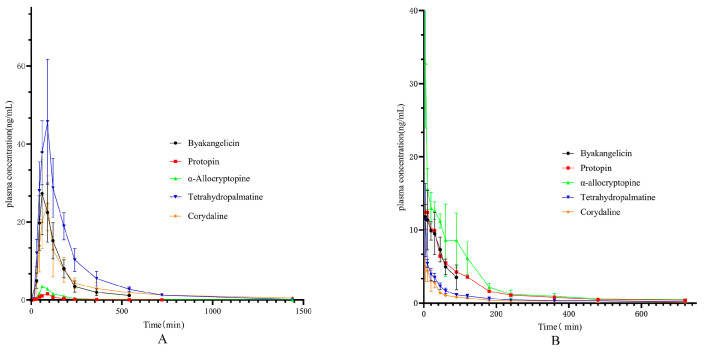
The mean plasma concentrations versus time profiles of protopin, α-allocryptopine, byakangelicin, tetrahydropalmatine, corydaline after oral administration (**A**) and intravenous administration (**B**) of single compound in beagle dogs (*n* = 6).

As shown in Figure 3, the plasma concentration–time curves of 5 Q-markers in YZTs in beagle dogs were developed after oral administration of YZTs. The free single com-pounds were also intravenously injected as control. The mean pharmacokinetics (PK) parameters of five components are displayed in Table 3. It could be found that absolute oral bioavailability of the Q-markers in YZTs ranged from 1.35% of protopin to 10.85% of tetrahydropalmatine. Based on the pharmacokinetic results, these Q-markers did not achieve satisfactory bioavailability, which corresponds to poor dissolution outcomes. Due to the specific TCMP preparation process, YZTs are prepared using medicinal extract and medicinal powder, leading to prolonged release time of Q-markers in the body and reduced absorption of active ingredients.

### 2.6. IVIVCs Developed for Q-Markers in YZTs

The best-fitting models of the dissolution curves of five active components in different medium were optimized (Table 4), which would serve as in vitro parameters for con-structing IVIVC models. The results of the UIR parameters shown in Table 5 were used for deconvolution calculations to obtain the absorption curves of oral components in the body. 

Table 6 shows the dissolution results and in vivo absorption correlation results of the five active components under different dissolution conditions. Since the paddle method and the rotation speed 75 rpm were usually used in the dissolution of TCMPs, the medium was studied comprehensively. Six different dissolution media, including 0.1 M of HCl, acetate buffer, phosphate buffer, FaSSIF-V2, water, and FaSSGF, were compared. The larger the correlation coefficient value of the model, the better fitting in vitro and in vivo correlation model. For protopin, α-allocryptopine, byakangelicin, tetrahydropalmatine, and corydaline, the best in vitro and in vivo correlations dissolution media was 0.1 M of HCL, FaSSGF, acetate buffer, acetate buffer, FaSSGF, respectively, with the correlation coefficients of 0.9981, 0.9992, 0.9993, 0.9993, and 0.9986. The internal verification results showed that the PE% of C_max_ and AUC of α-allocryptopine, byakangelicin, tetrahydropalmatine, and corydaline were less than 15%, indicating that internal validation of the model meets the requirements. However, the IVIVC model of correlation of protopin was not validated internally. In order to study the suitable conditions for in vitro dissolution of protopin, we further investigated the correlation between in vitro dissolution and in vivo absorption of protopin at different rotation speeds with 0.1 M of HCl as dissolution medium. The results showed that the maximum correlation coefficient between the in vivo and in vitro was 0.9991 at a rotational speed of 100 rpm, and the internal validation of PE% results were less than 15%.

**Figure 4:**
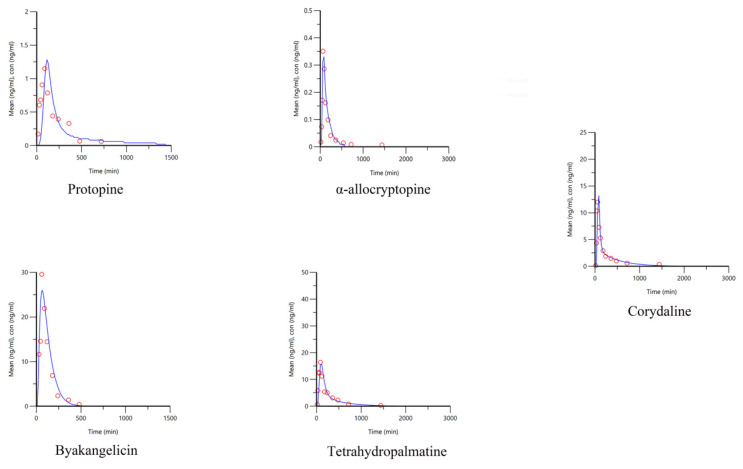
External validation of the IVIVC model for the five Q-markers in YZTs (dots are measured values, lines are predicted values).

In vitro dissolution data and in vivo pharmacokinetic data of another brand of YZTs were obtained for external validation. The dissolution data of the products in the identified media were input into the established IVIVC model to obtain the predicted blood concentration–time curve, and to compare the PE% between the predicted value and the actual value, as shown in Figure 4 and Table 7. The results showed that the identified dissolution conditions could accurately predict the pharmacokinetic profiles of different batches of YZTs. Therefore, the most specific in vitro–in vivo correlated dissolution conditions for α-allocryptopine, byakangelicin, tetrahydropalmatine, and corydaline are shown in Table 8.

### 2.7. Integrated IVIVC of Multiple Q-Markers in YZTs

**Figure 5:**
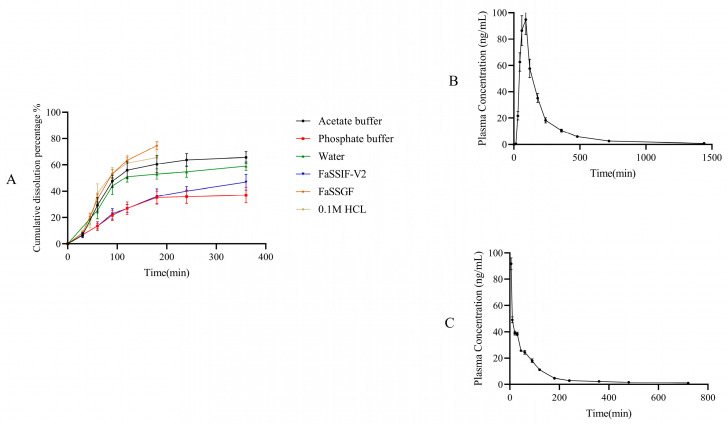
(**A**) In vitro integrated dissolution data of the BCS I Q-markers of YZTs in different medium including 0.1 M of HCl, acetate buffer, phosphate buffer, FaSSIF-V2, and FaSSGF; Oral (**B**) and Intravenous (**C**) pharmacokinetic integration results of the BCS I Q-markers in YZTs.

According to the results of the BCS classification study, all five Q-markers, i.e., tetra-hydropalmatine, corydaline, protopin, α-allocryptopine, and byakangelicin, were BCS I compound. Therefore, the integration of the dissolution data was obtained by calculating the dissolution and pharmacokinetic data of the five BCS Class I Q-markers according to the methods in Section 3.8.1 and Section 3.8.2. Figure 5A illustrates the dissolution fraction versus time profiles of BCS I Q-markers in various dissolution media from YZTs. The integrated pharmacokinetic profiles of the BCS I Q-markers are displayed in Figure 5B,C.

**Figure 6:**
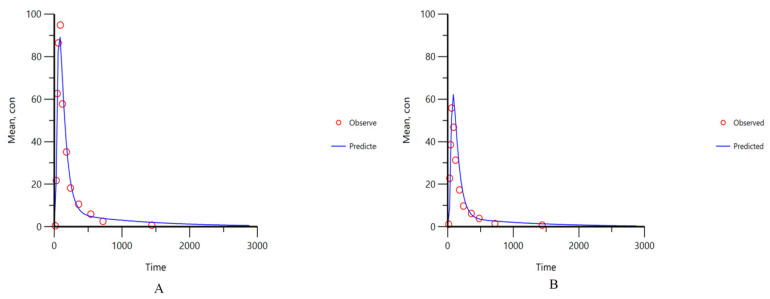
Validation results for dissolution conditions and IVIVC model ((**A**) for internal validation, (**B**) for external validation, dots are measured values, lines are predicted values).

The integrated dissolution and pharmacokinetic data were fitted to the in vitro dissolution data according to the steps of correlation model building in Section 3.7, respectively. The appropriate integrated dissolution fitting model (Table 9) was selected, and the in vivo absorption parameters (Table 10) were established to fit the absorption curves. Afterwards, the correlation model between integrated dissolution data under different conditions and integrated pharmacokinetic data was constructed separately; the results are shown in Table 11. For integrated BCS I Q-markers in YZTs, it could be found that the best IVIVC can be established when taking acetate buffer as dissolution medium. As shown in Figure 6, the results of internal and external validation demonstrated that the identified dissolution conditions could accurately predict the pharmacokinetic profiles of different batches of YZTs.

### 2.8. Application of Dissolution Evaluation Methods

**Figure 7:**
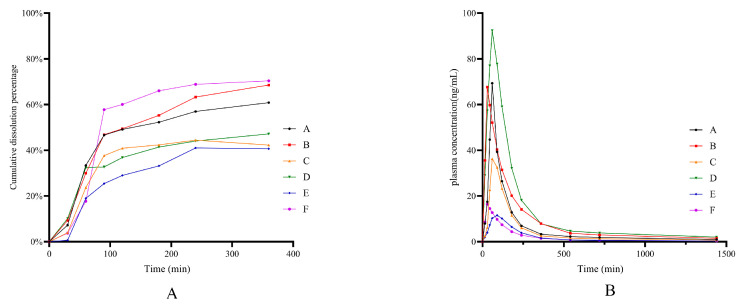
(**A**) In vitro integrated dissolution results of the BCS I Q-markers in different brands of YZTs; (**B**) In vivo integrated pharmacokinetic prediction results of the BCS I Q-markers in different brands of YZTs.

Five batches of YZTs named A, B, C, D, E, and F (A and B are the same brand but different lots, C, D, E, and F are different brands) were quantitatively analyzed, and the dissolution tests were carried out. Based on the dissolution curve, the pharmacokinetic curves of five batches of YZTs were predicted. The dissolution results and predicted concentration–time curves are shown in Figure 7. The AUC_0-∞_ and C_max_ of each component in each YZT are calculated in Table 12. It can be found that both in vitro dissolution profiles and predicted in vivo pharmacokinetic profiles of YZTs made from different companies are quite different. For the same brand made by the same company, the error between batches was the smallest. However, more obvious pharmacokinetic errors between different brands from different companies were observed. The differences may be due to differences in herb quality, tablet formulation, and preparation process, which have been ignored for a long time. The identified in vitro dissolution conditions can serve as an effective tool for comparing and evaluating the inner quality of different YZTs.

## 3. Materials and Methods

### 3.1. Chemicals, Reagents and Animals

**Figure 8:**
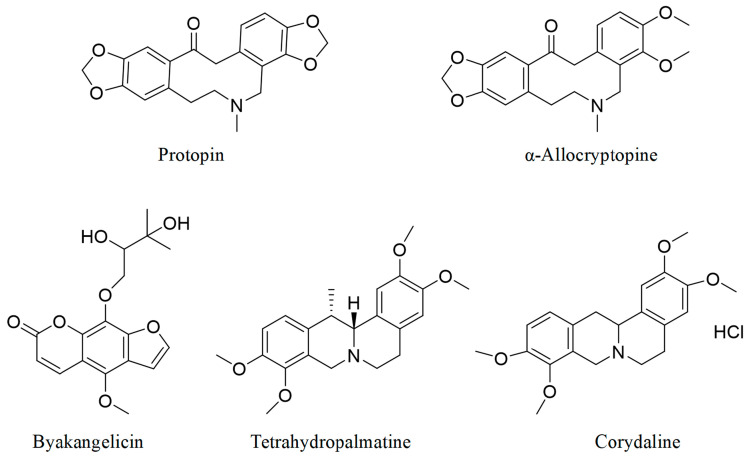
The chemical structures of protopin, α-allocryptopine, byakangelicin, tetrahydropalmatine, and corydaline.

The following reference standards were purchased from Chengdu Herbpurify Co., Ltd. (Chengdu China): tetrahydropalmatine (98.76%), corydaline (99.51%), protopine (99.02%), α-allocryptopine (98.79%), and byakangelicin (99.31%), and Figure 8 shows their chemical formulas. The internal standard (IS), warfarin, was obtained from J&K Scientific (Guangzhou, China). Acetic acid and ammonium acetate for HPLC were obtained from Aladdin (Shanghai, China), while acetonitrile for HPLC was obtained as the mobile phase from Fresh (Boston, MA, USA). Hydrochloric acid (37%), sodium acetate, acetic acid, potassium dihydrogen phosphate, and sodium hydroxide were purchased from Sinopharm Chemical Reagent Co., Ltd (Beijing, China). and were all of analytical grade. Water for experiments was prepared using a water purification system (Millipore, Milford, MA, USA). Yuanhu Zhitong tablets (YZTs) were purchased from Zhongjing Wanxi Pharmaceutical Co., Ltd. (Nanyang, China), Huluwa Pharmaceutical (Haikou China), Sunflower Pharmaceutical Group Co., Ltd. (Harbing, China), Guangxi Beibu Gulf Pharmaceutical Co., Ltd. (Qinzhou, China), and Yunnan Baiyao Group Co., Ltd. (Kunming, China), respectively.

Beagle dogs were purchased from Shanghai Jiaotong University College of Agricultural and Biological Sciences Experimental Practice Field Co (Shanghai, China). The animal experiments were approved by the Ethics committee for the care and use of laboratory animals at the School of Pharmacy, Fudan University (approval number of the Ethics: 2020-04-YJ-WJX-02).

### 3.2. Identification of Q-Markers

The previous study identified the active ingredients of YZTs and elucidated their molecular mechanisms by integrating chemical and biosynthetic analyses, drug metabolism [25], and network pharmacology [26]. Data mining methods including grey relational analysis (GRA) and least squares support vector machine (LSSVM) regression techniques were used to establish the correlation between the active ingredients and the efficacy and dosage efficacy of the drugs in multiple dimensions, and based on the results, the active ingredients of the YZTs were selected as tetrahydropalmatine, α-allocryptopine, protopine, corydaline, and byakangelicin. Based on the results, tetrahydropalmatine, α-allocryptopine, protopine, corydaline, and byakangelicin were selected as the Q-markers for YZTs [45].

### 3.3. Determination of Q-Markers by High-Performance Liquid Chromatography Method

The contents of quality control components were determined with high-performance liquid chromatography (HPLC) using a reverse column Agilent SB (4.6 × 150 mm, 3.5 μm). The mobile phase comprised a 10 mM ammonium acetate solution (pH 5.0 with acetic acid) and acetonitrile. A gradient elution procedure was employed with the following conditions: 0–10 min: 7% acetonitrile, 10–13 min: 15% acetonitrile, 17–20 min: 28% acetonitrile, 20–39 min: 30% acetonitrile, and 39–45 min: 35% acetonitrile. The flow rate was set at 1.5 mL/min, the sample volume was 10 μL, the temperature was ambient, and the detection wavelength was set at 280 nm.

### 3.4. BCS Classification of Q-Markers in YZTs

The Biopharmaceutical Classification System (BCS) categorizes compounds based on their solubility at the highest therapeutic dose and gastrointestinal permeability. The BCS classification of the Q-markers in YZTs, i.e., tetrahydropalmatine, α-allocryptopine, protopine, corydaline, and byakangelicin, was studied by measuring their solubility and permeability.

#### 3.4.1. Solubility of Q-Markers in YZTs

The solubility of the compounds was determined at physiologically relevant pH values (0.1 M of HCl, pH 1.2; acetate buffer, pH 4.5; and phosphate buffer, pH 6.8) as those in dissolution medium, by adding excess compounds to specific medium, vortexing for 30 s, and shaking for 24 h at 37 °C and 120 rpm using a constant temperature shaker. The supernatant was then diluted with an appropriate amount of methanol, and the concentration of each compound was determined using the HPLC method described in Section 3.2. The solubility was evaluated using the dose (D_0_) values [48], which describe the relationship between solubility and maximum dose strength according to Equation (1). Compounds with a D_0_ value lower than or equal to 1 are considered highly soluble.

(1) $$D_{0}=\frac{M_{0}}{V_{0}}/C_{S}$$

M_0_ was the highest dose of each component under single administration, it should be noted that since there is no sense of the maximum oral dose for the ingredients in herbal preparation, the content of each component in a single dose of the preparation is taken as the maximum administered dose. V_0_ = 250 mL (volume of water taken with the dose), and Cs is the minimum solubility at pH 1.2, 4.5, and 6.8 at 37 °C in mg/mL. 

#### 3.4.2. Permeability of Q-Markers in YZTs

The gastrointestinal permeability of the quality control components was measured using Caco-2 transport assay [49]. Caco-2 cells were inoculated onto 24-well transwell^TM^ plates at a density of 1 × 10^5^ cells per well. After 21 days of incubation, when the transepithelial electrical resistance (TEER) of the Caco-2 cells on the transwell^TM^ plate reached above 300 Ω·cm^2^, three different (high, medium, and low) concentrations of tetrahydropalmatine, α-allocryptopine, protopine, corydaline, and byakangelicin with cell inhibition less than 10% were subjected to transfer experiments. Different concentrations of the compounds were added to the apical (AP) side, and 100 µL of the liquid sample was aspirated from the basolateral (BL) side after 30, 60, and 120 min, and supplemented with the same volume of Hank’s solution. The HPLC method described in Section 3.2 was applied to determine the concentration of the Q-markers. The apparent permeability coefficient (P_eff_) was calculated according to Equation (2):

(2) $$P_{eff}=\frac{\left(dQ\right)/dt}{AC_{0}}$$

where dQ/dt is the amount of drug transported per unit time; A is the area of the transport membrane, which is 0.33 cm^2^ in this study. C_0_ is the initial concentration of the drug under test.

### 3.5. Dissolution Tests of Q-Markers in YZTs in Various Media

The dissolution of five Q-markers in YZTs was evaluated using the paddle method in accordance with the Chinese Pharmacopoeia. A popular brand of YZTs was chosen for dissolution experiment in six different media, including 0.1 M of HCl (pH = 1.2), acetate buffer (pH = 4.5), phosphate buffer (pH = 6.8), water (pH = 6.9), fasting simulated gastric fluid (FaSSGF), and fasting simulated intestinal fluid (FaSSIF-V2). The FaSSGF and FaSSIF-V2 were prepared as shown in Tables S5 and S6. The dissolution tests were conducted at a temperature of 37 ± 0.5 °C and a paddle speed of 75 rpm. Add 6 tablets to each dissolving cup, accompanied by 900 mL of dissolving solution, in 6 parallel aliquots, and samples of 3 mL were collected at 15, 30, 45, 60, 90, 120, 180, 240, and 360 min, respectively. Taking into account the gastric emptying time, the end time of dissolution measurement was 180 min when taking 0.1 M of HCl as dissolution medium. The dissolution medium was replenished after each sampling. The samples were filtered through 0.22 μm membrane and analyzed using the HPLC method described in Section 3.2. The chromatographic results were processed to calculate the concentration of each component at different time points, and the dissolution results were calculated using Equation (3) as follows:

(3) $$D=\frac{(C_{n}V_{1}+V_{2}\sum_{i=1}^{n-1}C_{n-1})\times 100\%}{M}$$

C_n_ is the drug concentration at time point n; V_1_ is the volume of dissolution medium in the dissolution cup; V_2_ is the volume of the sample removed at each time point; M is the total drug content.

### 3.6. Pharmacokinetics of Q-Markers in YZTs

#### 3.6.1. UPLC-MS/MS Conditions for the Determination of Q-Markers in the Blood

An ACQUITY UPLC system from Waters was used for chromatography separation, equipped with a Waters Cortecs C18 column (1.7 μm, 2.1 mm × 50 mm) operated at a flow rate of 0.30 mL/min. The column temperature and auto-sampler temperature were set as 45 °C and 4 °C, respectively. The mobile phases were 10 mM ammonium acetate solution (pH 5.0 by acetic acid) (A) and acetonitrile (B) and the gradient elution used was as follows: 30–39% B from 0–3.3 min, 39–95% B from 3.3–5.4 min, 95% B from 5.4–7.4 min, 95–30% B from 7.4–13.5 min, and 30% B from 13.5–16.0 min. The injection volume was 3 μL. The AB SCIEX Qtrap™ 6500 LC-MS/MS system with electron spray ionization (ESI) source in multiple reaction monitoring (MRM) mode was used for MS analysis. The detection was performed under the following conditions: source temperature (at set point) at 550.0 °C, positive ion mode, GS1 at 40 psi, GS2 at 40 psi, CUR at 45 psi, CXP at 15 V, DP at 100 V, and EP at 10 V. Quantitative analysis of analytes in MRM mode was used to determine the transition of precursor ions to specific product ions (*m*/*z*): tetrahydropalmatine, *m*/*z* 273.1 [M + H]+ → *m*/*z* 229.0 (collision energy, CE: 17 V), corydaline, *m*/*z* 273.1 [M + H]+ → *m*/*z* 229.0 (CE: 17 V), protopin, m/z 244.8 [M + H]+ → *m*/*z* 227.1 (CE: 23 V), allocryptopine, *m*/*z* 393.2 [M + H]+ → *m*/*z* 373.2 (CE: 17 V), and byakangelicin, *m*/*z* 389.2 [M + H]+ → *m*/*z* 363.1 (CE: 17 V). The data were processed using MultiQuant™ 2.1 software purchased from SCIEX.

#### 3.6.2. Preparation of Standard Solutions Samples

The standard solutions of tetrahydropalmatine, corydaline, protopin, α-allocryptopine, and byakangelicin were prepared by dissolving them in acetonitrile to obtain a mixed standard stock solution, which was further diluted with acetonitrile to prepare a series of different concentration gradient mixed standard solutions. An internal standard solution of 25 ng/mL was prepared from betamethasone solution (500 μg/mL) diluted with acetonitrile. Blank dog blood was collected through intravenous blood sampling, and plasma was obtained by centrifugation. To prepare the standard plasma sample, 25 μL of the mixed standard solution was added to 100 μL of blank plasma, and 400 μL of acetonitrile was added and vortexed for protein precipitation. The supernatant was acquired by centrifugation and blown dry with nitrogen. Then, 100 μL of acetonitrile was added to the dried supernatant, and the resulting solution was mixed to gain the standard plasma sample. Different mixed standard solution including a serial of concentrations of tetrahydropalmatine: 0.625–25 ng/mL, corydaline: 0.625–25 ng/mL, protopin: 0.1–4 ng/mL, α-allocryptopine: 0.1–4 ng/mL, byakangelicin: 18.75–750 ng/mL were applied to prepare standard plasma sample and quality control (QC) samples of plasma as follows: 1.25, 5, and 20 ng/mL for tetrahydropalmatine and corydaline, 0.2, 0.8, and 3.2 ng/mL for protopin and α-allocryptopine, and 37.5, 150, and 600 ng/mL for byakangelicin.

#### 3.6.3. Pharmacokinetic Study of the Q-Markers

For pharmacokinetic study, six beagle dogs were used and housed in a professional animal breeding room with a relative humidity of 55–60% and a temperature of 25 °C for one week. The experimental operation was carried out in accordance with ethical requirements for animal experiments and approved by the Institutional Animal Care and Use Committee of the School of Pharmacy, Fudan University. The dogs had free access to drinking water and were fasted for 24 h before the experiment. The dogs were randomly divided into two groups, one group receiving an oral dose of 12 tablets with brand A (tetrahydropalmatine 2.5 mg, corydaline 0.963 mg, protopin 1.896 mg, α-allocryptopine 0.917 mg, and byakangelicin 2.783 mg), while the other group were given an intravenous dose of 2 mL of normal saline containing tetrahydropalmatine 19.75 μg, corydaline 4.54 μg, protopin 168.062 μg, α-allocryptopine 297.16 μg, and byakangelicin 78.68 μg. Blood samples of 2 mL were taken at 15, 30, 45, 60, 90, 120, 150, 180, 240, 360, 480, 720, and 1440 min after dosing. Plasma samples were prepared by centrifuging the blood samples (4000 rpm, 5 min) and were detected using UPLC-MS/MS. The pharmacokinetic results were analyzed using WinNonlin.

### 3.7. Development of IVIVC for Q-Markers in YZTs

The models for calculating correlations between in vitro drug properties (e.g., dis-solution) and in vivo responses (e.g., absorption fraction) are available in the Phoenix (Version 8.1) WinNonlin IVIVC toolkit. Correlation modelling can be performed using the IVIVC Wizard in the software. Three following main steps are conducted to establish IVIVC for Q-markers.

#### 3.7.1. Fitting of In Vitro Dissolution Profiles

The Phoenix WinNonlin IVIVC Toolkit provides Hill, Weibull, and Makoid Banakar functions to fit the average dissolution of each component in each dissolved medium [50]. The best-fitting model was selected according to the highest correlation coefficient (R) and the smallest Akaike information criterion (AIC) value in the fitting results.

Hill:

(4) $$F_{diss,vitro}=\frac{F_{inf}\times t^{b}}{{MDT}^{b}+t^{b}}$$

Weibull:

(5) $$F_{diss,vitro}=F_{inf}\times (1-exp[-{\frac{t-t_{lag}}{MDT}]}^{b})$$

Makoid Banakar:

(6) $$F_{diss,vitro}=F_{max}\times ({\frac{t}{T_{max}})}^{b}\times exp[b\times (1-\frac{t}{T_{max}})]\;\;for\;t\leq T\_max$$

(7) $$F_{diss,vitro}=F_{max}\;\;for\;t>Tmax$$

where F_diss,vitro_ is the fitting fraction dissolved at time t, F_inf_ is the fraction dissolved at time infinity (fixed as 1), MDT is the average dissolution time (hours), F_max_ is the maximum in vitro dissolution value, *t_lag_* is the lag time, T_max_ is the time corresponding to the maximum in vitro dissolution value, and b is the shape parameters.

#### 3.7.2. Fitting of In Vitro Dissolution Profiles

The Phoenix WinNonlin IVIVC tool was utilized to evaluate the relationship between in vivo drug dissolution rate and blood drug concentration, which utilizes the Unit Impulse Response (UIR) function for this purpose. By using intravenous administration as the input for the unit impulse response, the influence of drug absorption can be eliminated. The obtained UIR function will be utilized for the deconvolution of pharmacokinetic data to obtain the curve of cumulative drug absorption over time [50].

(8) $$Y(t)=A\times exp[Alpha\times (t\times Ka-t_{lag})]$$

in which Y(t) is unit impulse response, A is the coefficient value, Alpha is the exponential value, Ka is the absorption rate constant, t_lag_ is the lag time.

#### 3.7.3. Establishment and Validation of IVIVC Model

To establish an IVIVC model, four linear correlation formulas are constructed by fitting dissolution curves under different dissolution conditions with in vivo cumulative absorption curves.

(9) $$F_{abs}=AbsScale\times Diss(T_{vivo})$$

(10) $$F_{abs}=AbsScale\times Diss(T_{scale}\times T_{vivo})$$

(11) $$F_{abs}=AbsScale\times Diss(T_{scale}\times T_{vivo}-T_{shift})$$

(12) $$F_{abs}=AbsScale\times (Diss(T_{scale}\times T_{vivo}-T_{shift})-AbsBase)$$

Among them, F_abs_ is the fraction absorbed in vivo, AbsScale is the correlation factor between scaled in vitro release and in vivo absorption, Diss is the fraction dissolved in vitro, T_vivo_ is the nominal in vivo experimental time, T_scale_ is the time scale factor related to the ratio between in vitro drug release time and in vivo absorption time, and T_shift_ is the time shift factor describing the lag time between in vivo absorption and in vitro dissolution. The results of correlation coefficients, Akaike Information Criterion (AIC), and Schwarz Bayes Criterion (SBC) in the IVIVC model were used as the basis for the selection of the best fit; the higher the adjusted correlation coefficient, the lower AIC and SBC were observed for the best-fit model. 

The dissolution data used to establish a correlation model were applied to predict pharmacokinetic data and calculate the predicted error (PE%) between the actual observed values and the model-generated predicted values. Since internal validation is a metric to evaluate the performance of IVIVC models, the selected most suitable model is then internally evaluated by comparing the prediction error (PE%). The calculation formula for PE% is as follows:

(13) $$PE\%=[V_{P}-V_{0})/V_{0}]\times 100\%$$

Among them, V_P_ is predicted value and V_0_ is measured value. According to FDA and EMA guidelines, in order to guarantee the accuracy of outcome prediction by IVIVC, the mean absolute PE of C_max_ and AUC must be less than or equal to 15%. 

In order to further apply the identified in vivo correlated dissolution conditions to different batches of YZTs, another brand of YZTs was selected for external validation of the dissolution conditions and IVIVC model results. The dissolution data of different brands in the identified media were input into the established IVIVC model to obtain the predicted blood concentration–time curve and to compare the PE% between the predicted value and the actual value.

### 3.8. IVIVC of Multiple Q-Markers in YZTs

Based on the BCS classification, the dissolution data and pharmacokinetic data of the Q-markers with the same BCS class were integrated to obtain dissolution and pharmacokinetic profiles, respectively, to simplify the dissolution evaluation method. The IVIVC of the integrated dissolution profiles under different conditions and the integrated pharmacokinetics model was developed with Phoenix software (Version 8.1). 

#### 3.8.1. Integration of In Vitro Dissolution Data

The Q-markers in YZTs were classified according to BCS according to their solubility and permeability. Based on the knowledge that the compounds in same BCS class have similar dissolution and absorption properties, the dissolution profiles of different Q-markers within a BCS class are integrated into one dissolution profile according to their content in the tablet. Specifically, the weight coefficient (Wi) of each component is calculated based on the ratio of its content to the total content of Q-markers. The integrated cumulative dissolution rate is obtained by multiplying the cumulative dissolution (C) of each monomer component at each time point by their respective weight coefficients and adding them together. The weight coefficient of each component and the cumulative dissolution rate of the entire category are calculated using Equations (14) and (15), respectively.

(14) $$W_{i}={\;N}_{i}/(N_{i1}+N_{i2}+N_{i3}+N_{i4}+N_{i5})$$

(15) $$C_{t}=W_{i1}\times C_{i1\;}+W_{i2}\times C_{i2}+W_{i3}\times C_{i3}+W_{i4}\times C_{i4}+W_{i5}\times C_{i5}$$

i represents tetrahydropalmatine, corydaline, protopin, allocryptopine, and byakangelicin, N represents the content of each component in the preparation, and C_t_ represents the integrated cumulative dissolution rate at each time point.

#### 3.8.2. Integration of In Vivo Pharmacokinetic Data

Based on the determination results of Q-markers, the total blood concentration of all the Q-markers in YZTs is obtained by adding the blood concentrations of each component at the same time point. The formula for calculating the concentration is as follows:

(16) $$C_{T}=C_{j1}+C_{j2}+C_{j3}+\ldots +C_{jn}$$

in which j represents tetrahydropalmatine, corydaline, protopin, α-allocryptopine, byakangelicin, respectively. CT is the comprehensive concentration in beagle after correction with the custom weight coefficient.

#### 3.8.3. IVIVC Establishment of Multiple Q-Markers

According to the steps to establish IVIVC in Section 3.7, the integrated in vitro dissolution data were fitted to the four dissolution models to select the best-fitting results. The UIR was calculated from the integrated multiple Q-markers venous data, and then cumulative in vivo uptake curves of the multiple Q-markers by inverse convolution were drawn. The correlation models between the integrated dissolution curves under different dissolution conditions and the in vivo cumulative absorption curves were established to explore the most relevant in vitro dissolution conditions. Both internal and external validation are crucial steps in the validation process to ensure the accuracy, robustness, and generalizability of the correlation models.

### 3.9. Application of Dissolution Evaluation Methods

Based on the multiple Q-markers dissolution evaluation method established according to the experimental results in Section 3.8, the dissolution curves of five brands of YZTs available in the market were determined under selected conditions, which was used to evaluate the quality of commercially available YZTs.

## 4. Conclusions

In this study, the dissolution conditions of YZTs with in vitro and in vivo correlation were determined, which can be used to predict the in vivo pharmacokinetics curves of YZTs from different manufacturers and batches, and thus provide a reliable guidance for evaluating the quality difference of the tablets. In view of the complex characteristics of traditional Chinese medicine components, this study applied quality markers, which have been identified as active components to represent the efficacy of YZTs, as indexes, and integrated the index components according to BCS classification, to simplify the determination of dissolution curve. Our study established reliable in vitro dissolution conditions for the quality control and evaluation of YZTs and laid a good foundation for the dissolution study for TCMP with many active components. 

The Q-markers of YZTs all belong to the BCS I class, which reduces the difficulty of dissolution method research. However, due to the complexity and diversity of the active ingredients in many TCMPs, different BCS components may be added as Q-markers. Therefore, the development of rapid, efficient dissolution conditions related to in vivo absorption remains a great challenge. 

## Figures and Tables

**Table 1:** Solubility and D_0_ of five Q-markers of YZTs in three dissolution media (*n* = 3).

Compounds	Content	M_0_/V_0_ (mg/mL)	Phosphate Buffer (mg/mL)	Acetate Buffer (mg/mL)	0.1 M HCl (mg/mL)	D_0_		
	(mg/per Tablet)					Phosphate Buffer	Acetate Buffer	0.1 M HCl
Protopin	0.1742 ± 0.0043	0.00418	0.535 ± 0.0172	5.403 ± 0.378	4.700 ± 0.384	0.007813	0.000774	0.000889
α-allocryptopine	1.1042 ± 0.077	0.0265	0.0447 ± 0.00217	0.0458 ± 0.00256	0.0487 ± 0.00408	0.592841	0.578603	0.544148
Byakangelicin	0.0975 ± 0.0013	0.00234	0.00763 ± 0.00127	1.291 ± 0.0874	2.662 ± 0.0560	0.306684	0.001813	0.000879
Tetrahydropalmatine	0.3017 ± 0.0027	0.00724	0.0197 ± 0.00187	2.666 ± 0.539	0.655 ± 0.0433	0.367513	0.002716	0.011053
Corydaline	0.2871 ± 0.0039	0.00689	2.385 ± 0.348	>2.385	>2.385	0.002889	>0.002889	>0.002889

**Table 2:** The results of Peff and BCS class of five Q-markers of YZTs (*n* = 3).

Compounds	M_0_/V_0_ (mg/mL)	D_0_			In Vitro Caco-2 Cell Permeability (cm/s)	BCS Type
		Phosphate Buffer	Acetate Buffer	0.1 M HCl		
Protopin	0.00418	<1	<1	<1	7.49 × 10^−6^ ± 8.03 × 10^−8^	BCS I
α-allocryptopine	0.0265	<1	<1	<1	1.34 × 10^−5^ ± 9.69 × 10^−7^	BCS I
Byakangelicin	0.00234	<1	<1	<1	8.95 × 10^−6^ ± 2.14 × 10^−7^	BCS I
Tetrahydropalmatine	0.00724	<1	<1	<1	3.76 × 10^−6^ ± 6.88 × 10^−8^	BCS I
Corydaline	0.00689	<1	<1	<1	3.94 × 10^−6^ ± 3.73 × 10^−7^	BCS I

**Table 3:** Pharmacokinetic parameters of five Q-markers of YZTs in beagle dogs.

Project	Protopin		α-allocryptopine		Byakangelicin		Tetrahydropalmatine		Corydaline	
	po.	iv.	po.	iv.	po.	iv.	po.	iv.	po.	iv.
T_1/2_ (min)	319.26	274.64	503.22	331.43	197.15	46.52	233.75	399.67	524.58	237.47
T_max_(min)	90	5	60	5	60	5	90	5	90	5
C_max_ (ng/mL)	1.63	12.44	3.51	32.8	27.31	11.81	45.7	11.36	24.79	5.01
AUC 0-t	205.15	1349.21	498.70	2155.16	3579.24	666.41	8130.23	592.23	4451.82	390.19
AUC 0-inf	232.08	1507.52	547.02	2373.11	3916.93	702.63	8199.72	652.18	4867.99	430.94
Vz/F (L)	1.64	0.22	10.95	0.17	0.06	0.06	0.03	0.68	0.13	0.66
Cl/F (L/h)	0.21	0.03	0.9	0.02	0.01	0.05	0.01	0.07	0.01	0.11
Bioavailability (%)	1.35		7.5		3.81		10.85		5.38	

**Table 4:** Best-fitted dissolution models for five Q-markers of YZTs in different dissolution medium.

Compounds	Rpm	Media	Model	Functions	Correlation	AIC
Protopin	75	0.1 M HCl	Weibull	y(t) = 0.70 × (1 − exp[−(t/1.10)^2.06])	0.9909	−25.65
		Acetate buffer	Hill	y(t) = (0.78 × t^2.71)/(1.10^2.71 + t^2.71)	0.9981	−39.54
		Phosphate buffer	Weibull	y(t) = 0.62 × (1 − exp[−(t/1.50)^1.63])	0.9989	−44.11
		FaSSIF-V2	Hill	y(t) = (0.77 × t^1.73)/(1.90^1.71 + t^2.71)	0.9918	−32.08
		Water	Hill	y(t) = (0.77 × t^4.39)/(1.04^4.39 + t^4.39)	0.9907	−29.83
		FaSSGF	Weibull	y(t) = 0.82 × (1 − exp[−t^1.01])	0.9993	−37.41
α-allocryptopine	75	0.1 M HCl	Hill	y(t) = (0.90 × t^5.14)/(0.90^5.14 + t^5.14)	0.995	−23.86
		Acetate buffer	Weibull	y(t) = 0.86 × (1 − exp[−(t/1.26)^1.97])	0.9982	−38.41
		Phosphate buffer	Weibull	y(t) = 0.60 × (1 − exp[−(t/1.29)^1.78])	0.9954	−37.12
		FaSSIF-V2	Hill	y(t) = (0.60 × t^4.44)/(1.09^4.44 + t^4.44)	0.989	−30.3
		Water	Hill	y(t) = (0.88 × t^3.38)/(1.16^3.38 + t^3.38)	0.9963	−32.43
		FaSSGF	Hill	y(t) = (0.76 × t^1.31)/(1.84^1.38 + t^1.38)	0.9812	−23.29
Byakangelicin	75	0.1 M HCl	Hill	y(t) = (0.45 × t^2.74)/(1.17^2.74 + t^2.74)	0.9939	−33.46
		Acetate buffer	Hill	y(t) = (0.35 × t^2.79)/(1.12^2.79 + t^2.79)	0.9963	−45.32
		Phosphate buffer	Hill	y(t) = (0.23 × t^2.73)/(1.05^2.73 + t^2.73)	0.9976	−53.53
		FaSSIF-V2	Hill	y(t) = (0.27 × t^3.67)/(1.12^3.67 + t^3.67)	0.9849	−39.47
		Water	Hill	y(t) = (0.28 × t^3.61)/(1.02^3.61 + t^3.61)	0.9464	−31.76
		FaSSGF	Hill	y(t) = (1.36 × t^2.00)/(1.68^2.00 + t^2.00)	0.9964	−30.34
Tetrahydropalmatine	75	0.1 M HCl	Hill	y(t) = (0.72 × t^4.77)/(0.97^4.77 + t^4.77)	0.9916	−23.57
		Acetate buffer	Hill	y(t) = (0.79 × t^2.93)/(1.07^2.93 + t^2.93)	0.9978	−37.45
		Phosphate buffer	Weibull	y(t) = 0.30 × (1 − exp[−(t/2.29)^2.34])	0.9914	−35.17
		FaSSIF-V2	Weibull	y(t) = 0.38 × (1 − exp[−(t/3.48)^1.91])	0.9894	−36.65
		Water	Hill	y(t) = (0.64 × t^2.53)/(1.06^2.53 + t^2.53)	0.9869	−30.74
		FaSSGF	Hill	y(t) = (0.81 × t^1.12)/(2.27^1.12 + t^1.12)	0.9962	−34.31
Corydaline	75	0.1 M HCl	Weibull	y(t) = 0.79 × (1 − exp[−(t/1.06)^2.00])	0.996	−29.57
		Acetate buffer	Weibull	y(t) = 0.71 × (1 − exp[−(t/1.23)^2.04])	0.9994	−48.93
		Phosphate buffer	Weibull	y(t) = 0.25 × (1 − exp[−(t/1.94)^2.82])	0.986	−34.66
		FaSSIF-V2	Weibull	y(t) = 0.15 × (1 − exp[−(t/3.14)^1.77])	0.9793	−45.72
		Water	Hill	y(t) = (0.53 × t^4.53)/(1.03^4.53 + t^4.53)	0.9784	−29.32
		FaSSGF	Hill	y(t) = (0.44 × t^0.98)/(3.25^0.98 + t^0.98)	0.9987	−46.79

**Table 5:** Best-fitted UIR parameters for five Q-markers of YZTs via Akaike Model.

Compounds	Model	Weighting	Parameters	Estimate	Correlation	AIC	SBC
Protopin	Akaike	1/(Y × Y)	T_lag_ (h)	0.44	0.9451	−27.92	−25.53
			Ka (h^−1^)	1.36			
			A1 (ng × mL^−1^)	0			
			alpha1 (h^−1^)	1.35			
α-allocryptopine	Akaike	1/Yhat × Yhat	T_lag_ (h)	0.67	0.9973	−10.54	−8.12
			Ka (h^−1^)	5.65			
			A1 (ng × mL^−1^)	0			
			alpha1 (h^−1^)	1.13			
Byakangelicin	Akaike	1/Yhat	T_lag_ (h)	0.45	0.9966	1.65	2.83
			Ka (h^−1^)	2.23			
			A1 (ng × mL^−1^)	0.02			
			alpha1 (h^−1^)	1.18			
Tetrahydropalmatine	Akaike	1/Y	T_lag_ (h)	0.40	0.9886	22.53	25.44
			Ka (h^−1^)	1.28			
			A1 (ng × mL^−1^)	0.04			
			alpha1 (h^−1^)	1.30			
Corydaline	Akaike	1/Y	T_lag_ (h)	0.44	0.9761	10.42	13.33
			Ka (h^−1^)	1.46			
			A1 (ng × mL^−1^)	0.05			
			alpha1 (h^−1^)	1.42			

**Table 6:** IVIVC models for five Q-markers of YZTs and their internal validation of AUC and C_max_.

Compounds	Media	Model	Correlation	AIC	SBC	Observed		Predicted		PE%	
						AUC	Cmax	AUC	Cmax	AUC	Cmax
Protopin	0.1 M HCl	Fabs = 1.72 × (Diss (1.30 × Tvivo − 9.99) − 0.0015)	0.9981	−406.74	−396.28	205.15	1.63	228.87	1.30	11.56	−19.98
	Acetate buffer	Fabs = 1.55 × (Diss (1.77 × Tvivo − 20.10) − 4.74×10^-6^)	0.9946	−297.35	−286.89	205.15	1.63	227.94	1.23	11.11	−24.49
	Phosphate buffer	Fabs = 1.93 × Diss (2.78 × Tvivo − 78.11)	0.9932	−274.85	−267.01	205.15	1.63	236.66	1.34	15.36	−17.99
	Water	Fabs = 1.75 × (Diss (1.30 × Tvivo − 0.32) − 0.084)	0.991	−247.09	−236.63	205.15	1.63	250.86	1.36	22.28	−16.7
	FaSSGF	Fabs = 1.37 × (Diss (0.867 × Tvivo − 0.844) − 0.01)	0.987	−311.80	−207.02	205.15	1.63	149.90	1.32	−26.93	−18.77
	FaSSIF-V2	Fabs = 1.83 × (Diss (2.78 × Tvivo − 20.10) − 0.033)	0.9885	−222.79	−212.33	205.15	1.63	239.86	1.19	16.92	−26.96
α-allocryptopine	0.1 M HCl	Fads = 1.19 × Diss (1.14 × Tvivo − 0.27)	0.9985	−440.34	−432.49	498.70	3.51	516.25	3.27	3.52	−6.93
	Acetate buffer	Fads = 1.24 × Diss (1.80 × Tvivo − 20.10)	0.997	−383.80	−375.96	498.70	3.51	519.30	2.95	4.13	−16.01
	Phosphate buffer	Fads = 2.15 × (Diss (2.15 × Tvivo − 30.90) − 0.10)	0.9821	−203.59	−193.13	498.70	3.51	617.09	3.40	23.74	−3.27
	Water	Fads = 1.25 × (Diss (2.77 × Tvivo − 61.27) − 0.017)	0.9981	−427.94	−417.48	498.70	3.51	537.15	3.31	7.71	−5.77
	FaSSGF	Fabs = 2.52 × (Diss (1.17 × Tvivo − 10.05) − 0.016)	0.9896	−376.36	−365.90	498.70	3.51	467.63	2.57	−6.23	−26.81
	FaSSIF-V2	Fads = 1.80 × Diss (1.62 × Tvivo − 10.13)	0.9981	−419.84	−411.99	498.70	3.51	515.76	3.25	3.42	−7.27
Byakangelicin	0.1 M HCl	Fabs = 2.42 × (Diss (1.64 × Tvivo − 10.05) − 0.012)	0.9977	−338.25	−327.79	3579.24	27.31	3714.64	25.49	3.78	−6.68
	Acetate buffer	Fabs = 2.93 × Diss (1.83 × Tvivo − 20.10)	0.9993	−456.33	−448.49	3579.24	27.31	3620.86	25.60	1.16	−6.29
	Phosphate buffer	Fabs = 4.47 × Diss (1.81 × Tvivo − 21.59)	0.9806	−89.17	−81.33	3579.24	27.31	3464.10	22.71	−3.22	−16.84
	Water	Fabs = 3.66 × (Diss (1.35 × Tvivo − 7.29) − 0.0053)	0.9754	−75.77	−65.31	3579.24	27.31	3439.06	21.77	−3.92	−20.30
	FaSSGF	Fabs = 1.03 × (Diss (0.46 × Tvivo − 12.56) − 0.046)	0.9736	−357.51	−347.04	3579.24	27.31	3697.35	24.55	3.30	−10.09
	FaSSIF-V2	Fabs = 3.70 × Diss (1.52 × Tvivo − 6.11)	0.9991	−425.45	−417.61	3579.24	27.31	3606.47	26.33	0.76	−3.61
Tetrahydropalmatine	0.1 M HCl	Fabs = 1.42 × Diss (0.98 × Tvivo)	0.9909	−260.66	−255.43	8130.23	45.70	8270.26	51.32	1.72	12.30
	Acetate buffer	Fabs = 1.30 × Diss (1.11 × Tvivo)	0.9993	−533.44	−528.21	8130.23	45.70	8301.00	42.28	2.10	−7.47
	Phosphate buffer	Fabs = 3.40 × Diss (1.93 × Tvivo)	0.9933	−301.81	−296.58	8130.23	45.70	8142.89	46.02	0.16	0.71
	Water	Fabs = 1.95 × (Diss (1.27 × Tvivo − 0.00082) − 0.108)	0.9843	−212.60	−202.14	8130.23	45.70	9510.40	44.41	16.98	−2.81
	FaSSGF	Fabs = 1.42 × (Diss (1.15 × Tvivo − 0.012) − 0.0084)	0.9991	−433.00	−422.55	8130.23	45.70	9588.79	40.25	17.94	−11.92
	FaSSIF-V2	Fabs = 2.77 × (Diss (2.78 × Tvivo − 0.00064) − 0.0029)	0.995	−327.91	−317.45	8130.23	45.70	8298.37	42.77	2.07	−6.41
Corydaline	0.1 M HCl	Fabs = 1.31 × Diss (1.07 × Tvivo − 9.25)	0.9962	−351.16	−343.32	4451.82	24.79	4588.14	22.91	3.06	−7.59
	Acetate buffer	Fabs = 1.46 × Diss (1.23 × Tvivo − 9.97)	0.9961	−349.97	−342.13	4451.82	24.79	4588.20	22.98	3.06	−7.29
	Phosphate buffer	Fabs = 4.18 × Diss (1.73 × Tvivo − 0.0022)	0.9937	−299.87	−292.03	4451.82	24.79	4509.00	24.89	1.28	0.40
	Water	Fabs = 1.95 × Diss (1.04 × Tvivo − 0.00073)	0.9856	−188.60	−180.75	4451.82	24.79	4453.68	22.13	0.04	−10.73
	FaSSGF	Fabs = 1.19 × (Diss (1.18 × Tvivo − 10.05) − 0.0136)	0.9968	−385.05	−374.59	4451.82	24.79	4812.75	22.74	8.11	−8.27
	FaSSIF-V2	Fabs = 7.47 × (Diss (2.78 × Tvivo − 7.71) − 0.00523)	0.9948	−318.66	−308.20	4451.82	24.79	4743.97	21.49	6.56	−13.31

**Table 7:** PE% of C_max_ and AUC in the external validation of Q-markers in YHTs.

	Predicted		Observed		PE%	
	AUC	C_max_ (ng/mL)	AUC	C_max_ (ng/mL)	AUC	C_max_
Byakangelicin	3754.39	25.75	3268.72	29.55	14.86	−12.85
α-allocryptopine	103.98	0.47	122.14	0.50	14.89	−6.01
Tetrahydropalmatine	3725.00	15.53	3596.97	16.40	3.56	−5.31
Corydaline	2143.49	12.35	2060.36	11.98	4.03	3.05
Protopine	218.10	1.26	205.15	1.15	9.77	11.21

**Table 8:** In vitro dissolution conditions for the best IVIVC model fits for Q-markers of YHTs.

Compounds	Method	Rotation Speed (rpm)	Media
Protopin	Paddle	100	0.1 M HCL
α-allocryptopine		75	0.1 M HCL
Byakangelicin		75	Acetate buffer
Tetrahydropalmatine		75	Acetate buffer
Corydaline		75	FaSSGF

**Table 9:** Best-fitted integrated dissolution models of BCS I Q-markers of YZTs in different dissolution conditions.

Compounds	Rpm	Media	Model	Functions	Correlation	AIC
BCS I Q-markers	75	0.1 M HCl	Hill	y(t) = (0.67×t^3.46)/(0.97^3.46 + t^3.46)	0.9932	−27.19
		Acetate buffer	Hill	y(t) = (0.65×t^2.84)/(1.07^2.84 + t^2.84)	0.9995	−50.62
		Phosphate buffer	Hill	y(t) = (0.39×t^2.33)/(1.34^2.33 + t^2.33)	0.9951	−40.47
		FaSSIF-V2	Hill	y(t) = (0.45×t^1.79)/(1.84^1.79 + t^1.79)	0.9968	−45.58
		Water	Hill	y(t) = (0.57×t^3.35)/(1.06^3.35 + t^3.35)	0.9912	−33.99
		FaSSGF	Hill	y(t) = (0.81×t^1.11)/(2.46^1.11 + t^1.11)	0.9996	−38.01

**Table 10:** Best-fitted UIR parameters for BCS I Q-markers of YZTs via Akaike Model.

Compounds	Model	Weighting	Parameters	Estimate	Correlation	AIC	SBC
BCS I Q-markers	Akaike	1/Y	A1 (ng × mL^−1^)	0.360	0.9953	15.55	18.46
			Alpha1 (h^−1^)	0.480			
			A2 (ng × mL^−1^)	0.0760			
			Alpha2 (h^−1^)	0.015			

**Table 11:** IVIVC models for the BCSI Q-markers of YHTs and their internal validation of AUC and C_max_.

Compounds	Media	Model	Correlation	AIC	SBC	Observed		Predicted		PE%	
						AUC	Cmax	AUC	Cmax	AUC	Cmax
BCS I Q-markers	0.1 M HCl	Fabs = 0.33 × (Diss (0.96 × Tvivo − 5.05) − 0.0012)	0.9932	−604.12	−593.66	16,507.52	94.83	17,734.50	91.22	7.43	−3.81
	Acetate buffer	Fabs = 0.34 × (Diss (1.20 × Tvivo − 6.19) − 0.0087)	0.9953	−640.24	−629.78	16,507.52	94.83	18,004.90	87.05	9.07	−8.20
	Phosphate buffer	Fabs = 0.69 × (Diss (1.57 × Tvivo − 4.38) − 0.057)	0.9833	−513.00	−502.54	16,507.52	94.83	19928.20	94.10	20.72	−0.78
	Water	Fabs = 0.38 × Diss (1.07 × Tvivo − 0.0059)	0.9810	−463.61	−455.76	16,507.52	94.83	17,005.65	81.66	3.02	−13.89
	FaSSGF	Fabs = 0.29 × (Diss (1.27 × Tvivo − 9.699) − 0.008)	0.9920	−587.31	−576.85	16,507.52	94.83	17,888.17	87.58	8.36	−7.65
	FaSSIF-V2	Fabs = 0.26 × (Diss (1.57 × Tvivo − 20.10) − 0.0068)	0.9949	−632.07	−621.61	16,507.52	94.83	17,887.37	85.07	8.36	−10.29

**Table 12:** Predictions of pharmacokinetics for different brands and probable error with different batches.

Brand Name	AUC	C_max_ (ng/mL)	PF%	
			AUC	Cmax
A (control subjects)	6096.27	34.62		
B	6214.88	33.82	1.95%	−2.31%
C	3033.97	18.15	−50.23%	−47.57%
D	8763.84	46.15	43.76%	33.30%
E	1361.92	5.77	−77.66%	−83.33%
F	1391.3	8.2	−77.18%	−76.31%

## Data Availability

Data sharing not applicable to this article as no datasets were generated or analysed during the current study.

## Supplementary Materials

The following supporting information can be downloaded at: https://www.mdpi.com/article/10.3390/ph17081065/s1, Table S1: Calibration curves and linear ranges of five Q-markers of YZTs; Table S2: Recovery of five Q-markers of YZTs in plasma (*n* = 6); Table S3: Precision and accuracy of five Q-markers of YZTs in plasma (*n* = 6); Table S4: Stability of five Q-markers of YZTs in plasma (*n* = 6); Table S5. Composition of the biorelevant media FaSSGF; Table S6: Composition of the biorelevant media FaSSIF-V2.
