# Supplementary material for: Establishment of Dissolution Test Method for Multi-Components in Traditional Chinese Medicine Preparations Based on In Vitro–In Vivo Correlation
Source: Pharmaceuticals (Basel). 2024 Aug 14;17(8):1065. doi: 10.3390/ph17081065 (PMC11359531; doi:10.3390/ph17081065)
Supplement: Supplementary file 1 [file pharmaceuticals-17-01065-s001.zip › pharmaceuticals-3101561-supplementary.pdf]

Supplementary Materials:

Table S1. Composition of the biorelevant media FaSSGF.

Composition	
Sodium chloride (μM）	80
Lecithin (μM)	20
Pepsin (mg/ml)	0.1
Sodium chloride (mM)	34.2
Hydrochloric acid qs	pH 1.6
Deionized water qs ad	11
pH	1.6
Osmolality (mOsm kg^{-1})	120.7±2.5

Table S2. Composition of the biorelevant media FaSSIF-V2.

Composition	
Sodium taurocholate (mM）	3
Lecithin (mM）	0.2
Maleic acid (mM)	19.12
Sodium hydroxide (mM)	34.8
Sodium chloride (mM)	68.62
pH	6.5
Osmolality (mOsm kg^{-1})	180±10
Buffer capacity (mmol l^{-1} ΔpH^{-1})	10

Table S3. Calibration curves and linear ranges of five Q-markers of YZTs

Compounds	Calibration curves	R^2	LLQC（ng/mL)	Range（ng/mL）
Protopin	Y=0.9727X-0.2319	0.9998	0.100	0.100-4.000
α-allocryptopine	Y=1.9122X-1.5453	0.9999	0.100	0.100-4.000
Byakangelicin	Y=3.9077X-1.4351	0.9992	18.750	18.750-750.000
Tetrahydropalmatine	Y=1.3125X-0.5506	0.9995	0.625	0.625-25.000
Corydaline	Y=0.8462X-0.3298	0.9997	0.625	0.625-25.000

Table S4. Recovery of five Q-markers of YZTs in plasma (n = 6)

Compounds	Concentration (ng/mL)	Recovery (%)		Matrix effect t(%)	
		MEAN	RSD	MEAN	RSD
Protopin	0.2	105.06	9.84	99.23	7.32
	0.8	90.34	8.13	93.42	8.44
	3.2	100.41	7.44	103.78	9.09
α-allocryptopine	0.2	102.94	9.63	90.39	8.93
	0.8	90.82	10.32	102.44	9.24
	3.2	101.66	10.93	97.49	3.76
Byakangelicin	37.5	98.99	6.94	99.37	6.38
	150	107.74	7.48	105.21	2.01
	600	85.39	3.22	108.37	10.35
Tetrahydropalmatine	1.25	109.93	6.83	94.38	7.44
	5	96.50	4.99	99.75	4.76
	20	98.47	11.27	91.39	5.29
Corydaline	1.25	106.48	3.85	101.35	10.86
	5	95.26	4.27	107.44	2.11
	20	99.57	5.51	101.62	3.86

Table S5. Precision and accuracy of five Q-markers of YZTs in plasma (n = 6)

Compounds	Concentration (ng/mL)	Intra-day			inter-day		
		Mean ± SD	Precision (%)	Accuracy (%)	Mean ± SD	Precision (%)	Accuracy (%)
Protopin	3.2	3.12±0.12	3.87	97.5	3.04±0.089	2.96	94.96
	0.5	0.496±0.013	2.6	99.19	0.51±0.022	4.44	101.81
	0.2	0.189±0.007	3.74	94.32	0.191±0.004	2.35	95.84
α-allocryptopine	3.2	3.06±0.14483	4.73	95.69	3.04±0.090	2.99	94.88
	0.5	0.52±0.0183	3.55	103.3	0.51±0.017	3.33	102.13
	0.2	0.19±0.0035	1.85	95.74	0.187±0.005	2.91	93.29
Byakangelicin	600	559.18±14.46	2.58	93.2	590.71±49.23	8.33	98.45
	150	168.47±6.90	4.1	112.31	177.98±11.78	4.23	113.87
	37,5	41.67±4.48	10.76	111.12	41.80±4.78	11.44	111.46
Tetrahydropalmatine	20	19.39±0.53	2.73	96.95	19.48±0.42	2.17	97.40
	5	5.21±0.14	2.74	104.2	5.10±0.22	4.39	102.00
	1.25	1.21±0.05	4.44	96.8	1.12±0.06	5.30	89.6.0
Corydaline	20	19.89±0.16	0.8	99.45	19.96±0.46	2.30	99.80
	5	5.38±0.19	3.54	107.6	5.41±0.14	2.67	108.2
	1.25	1.33±0.08	6.23	106.4	1.32±0.08	6.06	105.6

Table S6. Stability of five Q-markers of YZTs in plasma (n = 6)

Compounds	Concentration (ng/mL)	Relative content (%)		
		20℃-24h	6℃-6h	Freeze-thaw thrice
Protopin	3.2	101.63	103.43	101.56
	0.5	100.60	93.89	100.53
	0.2	96.07	99.33	96.00
α-allocryptopine	3.2	98.70	107.49	98.63
	0.5	101.06	100.95	100.99
	0.2	100.07	91.78	100.00
Byakangelicin	600	89.73	88.54	89.66
	150	93.39	93.99	93.32
	37.5	93.00	90.02	92.93
Tetrahydropalmatine	20	100.19	97.35	100.12
	5	102.60	99.79	102.53
	1.25	100.16	94.31	100.09
Corydaline	20	100.80	107.01	100.73
	5	102.35	93.89	102.28
	1.25	98.02	98.22	97.95
